# Draft Genome Sequences of Three Strains of Campylobacter jejuni Isolated from Patients with Guillain-Barré Syndrome in Bangladesh

**DOI:** 10.1128/MRA.00005-21

**Published:** 2021-04-29

**Authors:** Zhahirul Islam, Fahmida Habib Nabila, Asaduzzaman Asad, Ruma Begum, Israt Jahan, Shoma Hayat, Hubert P. Endtz

**Affiliations:** a Laboratory of Gut-Brain Signaling, Laboratory Sciences and Services Division (LSSD), icddr,b, Dhaka, Bangladesh; b Department of Medical Microbiology and Infectious Diseases, Erasmus University Medical Centre Rotterdam, Rotterdam, The Netherlands; University of Maryland School of Medicine

## Abstract

Campylobacter jejuni is the pathogen most commonly associated with Guillain-Barré syndrome (GBS). The present work describes the draft genome sequences of 3 C. jejuni strains, BD39, BD67, and BD75, isolated from stool specimens of patients with C. jejuni-triggered GBS using Illumina technologies.

## Abstract (precis)

Campylobacter jejuni is the pathogen most commonly associated with Guillain-Barré syndrome (GBS). The present work describes the draft genome sequences of 3 C. jejuni strains, BD39, BD67, and BD75, isolated from stool specimens of C. jejuni-triggered patients with GBS using Illumina technologies.

## ANNOUNCEMENT

Campylobacter jejuni is one of the prevailing diarrheal pathogens worldwide and causes Guillain-Barré syndrome (GBS) as a postinfection sequel (1, 2). Molecular mimicry between C. jejuni outer membrane lipooligosaccharides and host peripheral nerve gangliosides is widely postulated as the mechanism that triggers this autoimmunity (3–5). The interrelation between the microbial factors and host immunity that trigger autoreactivity is still unclear. Genome profiling of C. jejuni isolated from fecal samples from patients with GBS can unfold its genetic information and create a platform for comparison with other C. jejuni strains causing campylobacteriosis, which will help to reveal the detailed mechanisms and pathogenesis of GBS and other sequelae of *Campylobacter*-associated diarrhea. The study was approved by the institutional review board (IRB) of icddr,b, Bangladesh.

C. jejuni strains BD39, BD67, and BD75 were isolated from stool specimens from GBS patients using standard microbiological procedures (3). The C. jejuni isolates were enriched at 42°C for 48 h in blood agar with 5% sheep blood, and genomic DNA was extracted from the C. jejuni strains using the Wizard genomic DNA purification kit (Promega) following the manufacturer’s instructions (3). The DNA quality was determined using a NanoDrop spectrophotometer (Thermo Scientific, USA) and quantified using a Quantus fluorometer with the QuantiFluor ONE double-stranded DNA (dsDNA) system in order to fulfill sample quality requirements (quantity, 10 μg; concentration, *N*_50_, <200 ng/μg). Next-generation genome sequencing of the three C. jejuni strains was performed using the NextSeq 500 system (Illumina platform). The Illumina Nextera XT DNA library preparation kit (catalog number FC-131-1024) was used to prepare the sequencing library, and the NextSeq v2.5 reagent kit was used for sequencing. Quality checks on the paired-end sequencing reads (150 bp) were performed using FastQC v0.11.5 (6). Trimmomatic v0.36 was used for adapter trimming based on quality scores of Q30 with the following parameters applied: SLIDINGWINDOW:4:15, HEADGROUP:15, TRAILING:3, and MINLEN:36 (7). *De novo* assembly was performed using SPAdes v3.9.0 (8). Genome annotation was accomplished by the NCBI Prokaryotic Genome Annotation Pipeline (PGAP) v5.1 (9). CRISPRCasFinder (https://crisprcas.i2bc.paris-saclay.fr/CrisprCasFinder/Index) and the CRISPRTarget Web tool (http://crispr.otago.ac.nz/CRISPRTarget/crispr_analysis.html) were used to find and analyze clustered regularly interspaced short palindromic repeats (CRISPR) arrays. Default parameters were applied for all software unless otherwise specified.

The obtained genomic sequences had coverages of >200 for each strain. The guanine and cytosine (G+C) contents and genome sizes for these strains were found to be 31.8% to 32.3% and 1.59 Mbp to 1.77 Mbp (Table 1). From PGAP annotation, the C. jejuni strains have a total of 1,660 to 1,866 genes, including 46 RNAs for each strain. Moreover, CRISPR arrays were found in C. jejuni strains BD-39 and BD-67. The spacer sequences of these two strains target the protospacers of different plasmids and phages, but *Campylobacter phage* DA10 (GenBank accession number MN530981) was the common phage targeted by both strains (three protospacers targeted by one spacer sequence of BD-39 and two spacer sequences of BD-67). It is possible that the pathophysiology of C. jejuni is affected by the CRISPR-*cas9* system, which distinctively links C. jejuni bacteriophage defense, virulence, and GBS (11, 12).

**TABLE 1:** Genome features and accession numbers for Campylobacter jejuni strains BD-39, BD-67, and BD-75

								PGAP^*a*^ annotation						Read information			
Strain	LOS^*b*^^,^^*c*^	Penner type^*c*^^,^^*d*^	GenBank assembly accession no.	Genome size (bp)	G+C content (mol%)	No. of contigs	*N*_50_ (bp)	GenBank accession no.	Total no. of genes	No. of rRNAs			No. of tRNAs	SRA^*e*^ accession no.	No. of reads	Avg length (bp)	Genome coverage (×)
										5S	16S	23S					
BD-39	A	HS:19	GCA_003048165.1	1,599,909	32.3	35	145,897	NGUG00000000	1,660	1	1	1	40	SRR5363131	5,030,760	300	393
BD-67	B	HS:23	GCA_003048115.1	1,778,638	31.8	62	129,048	NGUF00000000	1,866	1	1	1	40	SRR5363132	11,984,930	300	842
BD-75	A	HS:55	GCA_003048185.1	1,651,474	32.0	18	183,845	NGUI00000000	1,734	1	1	1	40	SRR5363133	6,078,678	300	460

a PGAP, NCBI Prokaryotic Genome Annotation Pipeline.

b LOS, lipooligosaccharide.

c This information comes from Islam et al. (10).

d Penner heat-stable (HS) serotypes.

e SRA, Sequence Read Archive.

### Data availability.

All accession numbers are provided in Table 1.

## References

[1] Blaser MJ, Engberg J. 2008. Clinical aspects of Campylobacter jejuni and Campylobacter coli infections, p 99–121. *In* Nachamkin I, Szymanski CM, Blaser MJ (ed), Campylobacter, 3rd ed. ASM Press, Washington, DC.

[2] Guirado P, Paytubi S, Miró E, Iglesias-Torrens Y, Navarro F, Cerdà-Cuéllar M, Stephan-Otto Attolini C, Balsalobre C, Madrid C. 2020. Differential distribution of the wlaN and cgtB genes, associated with Guillain-Barré syndrome, in Campylobacter jejuni isolates from humans, broiler chickens, and wild birds. Microorganisms 8:325. doi:10.3390/microorganisms8030325. PMC7142995 32110976

[3] Islam Z, Sarker SK, Jahan I, Farzana KS, Ahmed D, Faruque ASG, Guerry P, Poly F, Heikema AP, Endtz HP. 2018. Capsular genotype and lipooligosaccharide locus class distribution in Campylobacter jejuni from young children with diarrhea and asymptomatic carriers in Bangladesh. Eur J Clin Microbiol Infect Dis 37:723–728. doi:10.1007/s10096-017-3165-7. 29270862

[4] Islam Z, Gilbert M, Mohammad QD, Klaij K, Li J, van Rijs W, Tio-Gillen AP, Talukder KA, Willison HJ, van Belkum A, Endtz HP, Jacobs BC. 2012. Guillain-Barré syndrome-related Campylobacter jejuni in Bangladesh: ganglioside mimicry and cross-reactive antibodies. PLoS One 7:e43976. doi:10.1371/journal.pone.0043976. 22952833 PMC3428305

[5] Yuki N. 2012. Guillain–Barré syndrome and anti-ganglioside antibodies: a clinician-scientist’s journey. Proc Jpn Acad Ser B Phys Biol Sci 88:299–326. doi:10.2183/pjab.88.299. PMC3422685 22850724

[6] Andrews S. 2010. FastQC: a quality control tool for high throughput sequence data. Babraham Bioinformatics, Babraham Institute, Cambridge, United Kingdom.

[7] Bolger AM, Lohse M, Usadel B. 2014. Trimmomatic: a flexible trimmer for Illumina sequence data. Bioinformatics 30:2114–2120. doi:10.1093/bioinformatics/btu170. 24695404 PMC4103590

[8] Bankevich A, Nurk S, Antipov D, Gurevich AA, Dvorkin M, Kulikov AS, Lesin VM, Nikolenko SI, Pham S, Prjibelski AD, Pyshkin AV, Sirotkin AV, Vyahhi N, Tesler G, Alekseyev MA, Pevzner PA. 2012. SPAdes: a new genome assembly algorithm and its applications to single-cell sequencing. J Comput Biol 19:455–477. doi:10.1089/cmb.2012.0021. 22506599 PMC3342519

[9] Tatusova T, DiCuccio M, Badretdin A, Chetvernin V, Nawrocki EP, Zaslavsky L, Lomsadze A, Pruitt KD, Borodovsky M, Ostell J. 2016. NCBI Prokaryotic Genome Annotation Pipeline. Nucleic Acids Res 44:6614–6624. doi:10.1093/nar/gkw569. 27342282 PMC5001611

[10] Islam Z, van Belkum A, Wagenaar JA, Cody AJ, de Boer AG, Tabor H, Jacobs BC, Talukder KA, Endtz HP. 2009. Comparative genotyping of Campylobacter jejuni strains from patients with Guillain-Barré syndrome. PLoS One 4:e7257. doi:10.1371/journal.pone.0007257. 19789649 PMC2748714

[11] Shabbir MAB, Tang Y, Xu Z, Lin M, Cheng G, Dai M, Wang X, Liu Z, Yuan Z, Hao H. 2018. The involvement of the Cas9 gene in virulence of Campylobacter jejuni. Front Cell Infect Microbiol 8:285. doi:10.3389/fcimb.2018.00285. 30177957 PMC6109747

[12] Louwen R, Horst-Kreft D, Boer AG, van der Graaf L, de Knegt G, Hamersma M, Heikema AP, Timms AR, Jacobs BC, Wagenaar JA, Endtz HP, van der Oost J, Wells JM, Nieuwenhuis EES, van Vliet AHM, Willemsen PTJ, van Baarlen P, van Belkum A. 2013. A novel link between Campylobacter jejuni bacteriophage defence, virulence and Guillain–Barré syndrome. Eur J Clin Microbiol Infect Dis 32:207–226. doi:10.1007/s10096-012-1733-4. 22945471

